# Of Issue Advocates and Honest Brokers: Participation of U.S. and German scientists in COVID-19 policy disputes

**DOI:** 10.1177/09636625251371565

**Published:** 2025-10-17

**Authors:** Nils Bienzeisler

**Affiliations:** Karlsruhe Institute of Technology (KIT), Germany

**Keywords:** politicization of science, science-policy interface, scientific expertise

## Abstract

The study examines the intersection of science and politics by analyzing the involvement of *N* = 205 U.S. and *N* = 174 German scientists in policy disputes during the COVID-19 pandemic. I investigate how scientists integrate themselves into policy disputes. Through a survey, I identify four groups of scientists with specific self-images regarding their roles in policy disputes: *Moderate Mainstreamers, Issue Advisors, Issue Advocates, and Honest Brokers*. Furthermore, the findings reveal differences in how these groups of scientists perceive the importance of science in policy-making: Particularly U.S.-based *Issue Advocates* wish for science to direct policy-making. In addition, I find that pandemic researchers overwhelmingly do not support political causes by selectively communicating political advice. I present empirically evidence that pandemic researchers sought to clarify the relevance of research during the pandemic, but did not attempt to distort policy disputes dishonestly.

During the COVID-19 pandemic, the involvement of scientists in *policy disputes* sparked controversy (cf. Nölleke et al., 2023). Policy disputes are public conflicts over how societies respond to issues through collective actions, commonly referred to as *policies* (cf. Palonen, 2003). The controversy was particularly evident in the public debates over containment measures, where individual freedoms and public health interests clashed. In April 2021, for example, scientists called for a unified effort to drastically lower SARS-CoV-2 infections across Europe (Priesemann et al., 2021). Meanwhile, in the United States, scientists called for a commission to analyze the country’s lax response to the COVID-19 pandemic (Chyba et al., 2021). Such position statements reignited a long-standing debate over the involvement of scientists in political matters with some scholars criticizing their peers for being overly politically engaged (e.g. Bogner, 2021), while others advocate for such involvement (e.g. Utz et al., 2022). Scientists were even criticized for aligning their policy advice with their own policy preferences (e.g. Hirschi, 2021).

Amid the controversial debates, it is not surprising that scientists researching the COVID-19 pandemic struggled to define their public role (cf. Biermann and Taddicken, 2024; Marcinkowski et al., 2025; Nölleke et al., 2023; Royan et al., 2023). Theoretical work suggests that scientists approach policy disputes in various ways: some impartially outline policy options, while others advocate for specific ones (Schmid-Petri et al., 2022; Pielke, 2007). The latter effectively join *politics*, the struggle for societal power and influence, to affect *policy-making*, the translation of policy disputes into, for example, legislation. Yet there is limited evidence on how scientists in politically charged fields perceive their involvement in policy disputes or if they even join politics (cf. Christensen, 2021). Some studies suggest that scientists tend to favor impartial advice and distance themselves from policy disputes (e.g. Tøsse, 2013; Wilke and Morton, 2015), while others suggest the opposite (e.g. Alinejad and van Dijck, 2022; Cologna et al., 2021; Graminius, 2023; Laing et al., 2022). This emphasizes the need for an exploration of how scientists integrate themselves into policy disputes. Moreover, it remains unclear how scientists in different political settings responded to policy disputes.

This study examines U.S. and German pandemic scientists during the final phase of the COVID-19 pandemic. Both countries represent different media ecosystems and degrees of political polarization, with the United States exemplifying high polarization, in contrast to the less polarized context of Germany (Brüggemann et al., 2014; Reiljan, 2020). I conducted a survey focusing on pandemic scientists’ beliefs, policy preferences, and strategies for disseminating policy advice, particularly in policy disputes about containment measures. The study aims to categorize pandemic scientists based on their beliefs. Moreover, I investigate whether U.S. and German pandemic scientists aligned their policy advice with their policy preferences.

Altogether, I identify four distinct groups of pandemic scientists, each characterized by particular beliefs regarding their role in policy disputes: Moderate Mainstreamers, who feel ambivalent about their role; Issue Advisors, who prefer to guide policy disputes as neutral experts; Issue Advocates, who actively engage in politics; and Honest Brokers, who refrain from asserting epistemic authority over policy disputes. I demonstrate that each group exhibits distinct characteristics, depending on the national context. However, I find that pandemic researchers do not support political causes through selective communication of policy advice.

## Scientists as public experts

Beyond their primary role in research, scientists engage in *science communication*, in the broadest sense publicly conveying scientific information (Scheufele, 2014). In addition, scientists provide knowledge to policymakers and laypeople (Jasanoff and Wynne, 1998). They respond to unanswered questions and propose solutions. In this domain, they operate as *public experts* (Peters, 2014). Public experts are typically asked to provide *policy advice*, that is, to assess how proposed policies might impact identified societal priorities, such as public health (Pielke, 2007: 30–36). They may support laypeople in decision-making contexts and expand options with their policy advice, for example in uncertain or risky situations (cf. Grundmann, 2017). Being recognized as a public expert requires acknowledgment from laypeople, as individuals seek expert advice only when they deem their judgment superior (Eyal, 2019; Grundmann, 2017; Peters, 2014). In knowledge societies, scientists and other knowledge professions are often naturally part of this group (Grundmann, 2017).

Communicating policy advice allows scientists some leeway. Pielke (2007: 14–21), for example, posits that scientists are characterized by their different approaches to the role as public experts: first, Pure Scientists focus exclusively on their research, with little regard for the application of their findings under the assumption of minimal interaction between science, politics, and society. Second, Science Arbiters maintain a distance from political involvement and provide policy advice solely upon request. Third, Issue Advocates participate in policy disputes by not only communicating policy advice but also supporting specific agendas and engaging in politics. Fourth, Honest Brokers also participate in policy disputes, but present various courses of action and their implications. Pielke (2007: 18) notes that Honest Brokers refrain from pursuing a specific political agenda and thus do not engage in politics.

However, the validity of this typology remains contested (e.g. Brown, 2008; Durant, 2023). For example, it seems implausible to me that Science Arbiters remain passive in the face of pressing issues, such as the COVID-19 pandemic. Moreover, scientists’ perceptions of the role as public expert often appear rather fuzzy and contradictory. Reflecting on this, I outline an alternative approach in the following section.

## Scientific identities and self-images of scientific experts

In policy disputes, scientists demonstrate various approaches to their role as public experts: plausibly, a scientist presenting a suitable solution in policy disputes differs from a scientist offering cautious policy advice, perhaps highlighting uncertainties. The various approaches scientists take as public experts can be understood as embodying different collective identities that result in diverse self-images.

Jasanoff (2015) conceptualizes *collective identities* as internalized perceptions and meaningful narratives that communities develop to define who they are and what they aspire to be, drawing on shared values and beliefs. For example, scientists view themselves as a distinct group, set apart from laypeople (Besley and Nisbet, 2013; Cook et al., 2004). Collective identities are shaped by institutional contexts and social interactions, and they evolve over time (Ashforth et al., 2008; Fischer and Schmid-Petri, 2023).^1^ Collective identities form individual *self-images* that locate people in the world (Foucault, 1998; Jasanoff, 2010). Self-images are internalized perceptions and meaningful narratives individuals hold about themselves. They structure how individuals see themselves and finally guide their actions (Foucault, 1998). Self-images may influence how scientists engage in policy disputes.

As scientists often struggle to define their public role (cf. Alinejad and van Dijck, 2022; Banse et al., 2025; Biermann and Taddicken, 2024; Laing et al., 2022; Nölleke et al., 2023), I believe that scientists harbour ambivalent self-images. I propose that scientists’ self-images related to policy disputes align along three key dimensions: political involvement, scientific neutrality, and epistemic authority.

### Political involvement

Research indicates that scientists vary in their political involvement—that is, in whether they take part in *politics* much like ordinary citizens. Citizens typically do so by standing up for their convictions or building majorities to advance their policy goals. In this regard, some scientists are deeply engaged (e.g. Alinejad and van Dijck, 2022; Biermann et al., 2023). Interview studies show that some scientists do not fully recognize their political activism, while others maintain a distance (Laing et al., 2022). Climate scientists detach themselves from politics (Alinejad and van Dijck, 2022; Tøsse, 2013), yet expect their peers to advocate (Cologna et al., 2021).

In a qualitative interview study, Alinejad and van Dijck (2022) highlight the contrasting views of two climate scientists, Peter and Bart, illustrating the debate. Peter sees political participation as integral to scientific inquiry, while Bart supports a more “traditional” view, where scientists inform policy disputes but stay out of politics. Both scholars, however, acknowledge the importance of each other’s public engagement. This suggests that scientists have conflicting self-images concerning their involvement in policy disputes and whether they should engage in politics. Empirical indicators can be seen in how strongly scientists stand up for their convictions, challenge hegemonies, or join together with other citizens.

### Scientific neutrality

Scientists also differ in how strongly they claim neutrality in policy disputes—that is, how far they distance themselves from politics and ideologies that uphold and validate the interests of a particular social group (cf. Laing et al., 2022; Post, 2013). While political involvement is about whether scientists engage in politics, scientific neutrality is about proving distance to politics. In the extreme, this means refraining even from policy advice (cf. Pielke, 2007).

The concept of scientific neutrality is central to the scientific community, since scientists are expected to be impartial (Dietz, 2013). More importantly, neutrality is connected to the formation of scientific facticity (Jasanoff, 2010). Scientific facticity involves the idea that scientific facts are shaped by historical negotiations, shared understandings, and values. Scientific truths are not just discovered; they are influenced by how society views them over time. As Jasanoff (2010) elucidates, scientific truths are co-constructed through interactions between science and politics, where the boundary between political relevance and scientific facticity is often politically motivated. Thus, maintaining a neutral stance is crucial for scientists to distinguish their claims from political actors (Jasanoff, 1994: 1).

Empirical findings indicate that scientists see themselves as impartial, valuing independence from practical considerations (Post, 2013). Graminius (2023) finds that scientists view activities like open letter communication as part of their professional self and thus apolitical. They often consider their participation in policy disputes aligned with the institutional values of science, allowing them to maintain a sense of neutrality while offering policy advice or even engaging in politics. However, impartiality can sometimes blur in the face of societal controversies (Bromley-Trujillo et al., 2014; Cologna et al., 2021; Post, 2016; Post and Ramirez, 2018). Thus, scientists attempt to demonstrate their scientific neutrality, even while engaging in politics much like ordinary citizens. Whether scientists see themselves as politically involved or neutral therefore may seem like a clear contrast, but in practice, the relationship is not straightforward.

Alinejad and van Dijck (2022) further explore the differing views of Peter and Bart on scientific neutrality. Peter, the politically engaged scientist, challenges neutrality, whereas Bart advocates for scientists’ impartiality. Considering the contrasting views of scientists, self-images centered on scientific neutrality may contribute to divisions within the scientific community. Empirical indicators of scientific neutrality can be seen in how consistently scientists refrain even from policy advice and emphasize their independence.

### Epistemic authority

Although scientific neutrality and political involvement are pivotal elements, they do not fully capture the complexities of scientists’ self-images. A scientist’s policy advice is meaningful only when coupled with the recognition of their epistemic authority (Eyal, 2019; Jasanoff and Wynne, 1998; Peters, 2014). Epistemic authority refers to scientists’ ability to define and disseminate knowledge within their field (Eyal, 2019: 26–29). For instance, young climate scientists see themselves as detached from policy-making and view their knowledge as junior. Therefore, they are less likely to share their expertise (van Eck, 2023). This illustrates that the acknowledgment of one’s own knowledge is a prerequisite for scientists to communicate publicly. The importance of epistemic authority is evident in how scientists view their knowledge as superior to the public’s understanding (Besley and Nisbet, 2013; Simis et al., 2016).

To illustrate this point with the example from earlier discussions, I return to Peter and Bart, as described by Alinejad and van Dijck (2022). Despite their differing views on political engagement and scientific neutrality, both senior scientists likely agreed on their own epistemic authority within their fields, potentially granting them a recognized position to influence policy disputes. Empirical indicators of epistemic authority can be seen in how strongly scientists position their expertise as a basis for determining appropriate policies.

In summary, the referenced studies highlight that scientists ponder their role in policy disputes: political involvement concerns their engagement in politics, scientific neutrality highlights their distance from it, and epistemic authority emphasizes their confidence in their knowledge and expertise. Building on this understanding, I ask: *How do pandemic scientists conceive of themselves in policy disputes (RQ1)?* In other words, what self-images do they hold?

## Scientists as advocates

When scientific experts extend their policy advice beyond sharing policy options, they venture into *advocacy.* Advocacy refers to the support of particular causes and represents a shift from scientists’ traditional role as neutral informers to participants in politics (Pielke, 2007: 135). Studies have shown that some scientists advocate for environmental and health policies (Jahng and Lee, 2018; Leidecker-Sandmann and Lehmkuhl, 2022). For example, scientists publicly supported mask mandates during the COVID-19 pandemic and called for political action (Biermann and Taddicken, 2024; Biermann et al., 2023, 2024).

Scientific advocacy faces a dilemma: while science may indicate a health crisis, opinions vary on prioritizing rigorous health measures versus other interests (cf. Angeli et al., 2021). This echoes Weber’s (1904) notion of the “fact-value” dichotomy, illustrating that personal values, which are subjective, stand apart from scientific facts, which aim for truth. Thus, scientific facts can only be seamlessly translated into policy-making when there is agreement on desired outcomes and minimal uncertainties (cf. Pielke, 2007: 22). However, scientists tend to view the intersection of science and politics as a straightforward, linear process, believing that policies can be deduced from policy advice without considering values (Alinejad and van Dijck, 2022; Calice et al., 2023; Simis et al., 2016). Scholars have therefore criticized the tendency of scientists to overestimate the role of science in policy-making (Bogner, 2021; Hirschi, 2021; Pielke, 2004). Scientists harboring political ambitions may mistakenly assume that science clearly reveals the best course of action and wish for science to direct policy-making. Thus, scientists like Peter, who hold political self-images, may find themselves particularly inclined to see the intersection of science and politics as linear. Hence, I ask: *How are pandemic scientists’ self-images related to the extent to which they wish science to direct policy-making (RQ2)?*

Research indicates that scientists being advocates make strategic decisions, sometimes aligning their policy advice with their policy preferences. Scientists might, for example, selectively emphasize certain data or predictions to sway public opinion toward specific actions (Post, 2016; Post and Ramirez, 2018). This reflects “stealth advocacy,” where scientists subtly influence policy disputes while maintaining an appearance of being impartial (Pielke, 2007: 92–96). Therefore, I *expect that pandemic scientists align their willingness to communicate policy advice with their policy preferences (H1).*

Given the interplay between behaviors and perceptions, I assume that scientists’ communicative manners are influenced by their self-images. This could be especially true for scientists harboring political ambitions (cf. Pielke, 2004). Thus, scientists like Peter, who hold political self-images, may sway public opinion using their epistemic authority. Therefore, I hypothesize that *pandemic scientists who blur the boundaries between science and politics are more inclined to align their willingness to communicate policy advice with their policy preferences (H2).*

As I can assume that scientists’ involvement in policy disputes is associated with the political landscape, I further explore *how the national context affects pandemic scientist’s participation in policy disputes* (RQ3). Different media ecosystems and degrees of political polarization are pertinent, with the United States exemplifying high polarization, in contrast to the less polarized context of Germany (Brüggemann et al., 2014; Reiljan, 2020). This distinction is particularly evident in the polarized COVID-19 debates in the United States compared with Germany (cf. Levin et al., 2023; Schmidt, 2023).

## Study design

Between the 21st of December 2022 and the 30th of January 2023, I surveyed scientists from the United States and Germany who published papers on COVID-19 in medical journals (hence pandemic scientists). At the start of the year 2023, the COVID-19 pandemic was nearing its conclusion, but uncertainty persisted. In both the United States and Germany, policymakers had to balance the need for containment measures with the observed seasonal trends in COVID-19 cases, hospitalizations, and reopening of society (Wiemken et al., 2023). A contentious question during this period was whether the COVID-19 situation should be regarded as a pandemic or an endemic. Linked to this question were differing positions on containment. In this climate, the study investigates how pandemic scientists communicate in policy disputes.

To examine the first and second research question, I surveyed the participants for their self-images and their wish for science to direct policy-making. Moreover, I implemented a between-subject experiment to test the first and second hypothesis. In this experiment, I presented three research findings and measured how important respondents found communicating related policy advice. I also explored differences between U.S. and German scientists.

All participants received information about the research before participating and gave informed consent. The study was approved by the *Institutional Review Board of the Karlsruhe Institute of Technology (KIT)*.

### Sample

I conducted an a priori power analysis to determine the necessary sample size for an analysis of variance (ANOVA) with three factors. The analysis estimated that a sample of 169 participants would provide 80% power at an alpha level of .05, assuming a minimally acceptable mediocre effect size of *f²* = .25 in each national context.

Next, I sourced the samples from the United States and Germany by accessing publicly available data of authors publishing in relevant scientific journals. To do so, I determined each five publication outlets with the highest impact in 12 medical research fields (e.g. Epidemiology, Virology) based on the Google H5 and the Web-of-Science-Index. Subsequently, I extracted all studies from the Web of Science database from 2020 until the 20th of December 2022 that contained one of four keywords (“COVID-19,” “Corona Virus,” “Corona,” or “SARS-CoV-2”). I selected all corresponding authors affiliated with a U.S. or German university or research facility according to the entry in the database. I excluded authors not affiliated with medical research institutions, such as those connected to government health departments. In the end, 244 U.S. (recruitment rate of 6%) and 192 German scientists (recruitment rate of 9%) participated in the survey.^2^ After quality checks and data cleaning to exclude scientists in non-medical fields (e.g. science communication), the sample size decreased to 205 U.S. and 174 German pandemic scientists included in the analysis.

U.S. pandemic scientists consisted of 42% females (cf. Supplemental Material 1). The median age was between 50 and 54 years.^3^ Most participants were professors (67%), followed by assistant professors (18%). They indicated that their research was primarily in public health (31%), psychology (11%), and immunology (9%). The sample for Germany consisted of 44% females. The median age was between 45 and 49 years. Most participants were postdoctoral researchers (40%), followed by regular professors (37%). They were primarily in public health (15%), psychology (15%), and virology (14%).

### Survey experiment

I presented each participant with one of three scenarios about a research project on SARS-CoV-2. The materials were slightly varied to evaluate pandemic scientists’ views on the appropriateness of using different scientific findings for policy advice. The first scenario depicted the virus as widespread with corresponding immunity, suggesting an endemic situation; the second scenario emphasized that the situation was *not* endemic (cf. Supplemental Material 2). This detail was a major point of contention at the time of the survey—Germany was debating the continuation of face mask mandates, while the United States faced renewed disputes over containment measures amid a seasonal spike (cf. Wiemken et al., 2023). A control group received a neutral description that only referenced the publication of research findings. The experimental groups were comparable in terms of demographics and participants’ political stance.^4^ In each scenario, participants later advised colleagues on communication decisions—though framed as counseling others, this serves as a proxy for each respondent’s own willingness to communicate policy advice in a policy dispute.

### Measures

#### Self-images

I developed a scale to assess how scientists integrate into policy disputes. I asked respondents, “How do you personally contribute to societal debates in your field?” and provided statements to rate on a 7-point Likert-type scale (1 = do not agree at all; 7 = absolutely agree). The scale was structured into three dimensions with each containing three items to measure self-images related to political involvement, scientific neutrality, and epistemic authority (cf. Table 1). The items covering political involvement concerned engagement in politics (“fighting,” “joining,” and “taking political action”). The items on scientific neutrality captured distance from politics (“avoiding conflict” and “maintaining independence”), with one extreme reflecting refusal to offer even policy advice. The items on epistemic captured how strongly scientists viewed their knowledge and expertise as a legitimate basis for policy-making (“demonstrating a way forward,” “implementing appropriate measures,” and “justifying necessary steps”). A principal component analysis (PCA) with varimax rotation revealed that each three items load on one of three factors, accounting for 68% of the variance (χ^2^(36) = 1040.40; *p* < .001; KMO = .77; Supplemental Material 3 for factor loadings). The items were used for segmentation and averaged to enable post hoc analyses (political involvement: *Cronbach’s α* = .79; scientific neutrality: *Cronbach’s α* = .73; epistemic authority: *Cronbach’s α* = .74).

**Table 1.** Survey items unrelated to scenarios.

*In societal debates, I. . .*	Self-images			
	U.S. Scientists		German Scientists	
	*M*	*SD*	*M*	*SD*
. . .fight for my political convictions.	3.84	1.76	4.11	1.73
. . .take political action for social change.	3.51	1.82	3.89	1.95
. . .join with others to build majorities for meaningful political action.	3.30	1.87	3.53	1.85
. . .take care not to give political advice.	3.89	1.77	3.95	1.84
. . .avoid getting involved in political conflicts whenever possible.	4.01	1.82	4.00	1.87
. . .maintain neutrality and independence from conflicting parties.	3.98	1.90	3.89	1.83
. . .strive to demonstrate to politics and society the best way forward with my scientific findings.	4.92	1.60	5.15	1.62
. . .use my expertise to work toward the implementation of scientifically appropriate measures.	4.89	1.69	5.27	1.59
. . .use my knowledge as an expert to justify necessary steps.	5.48	1.35	5.50	1.30
	Wish for science to direct policy-making			
	*M*	*SD*	*M*	*SD*
Science should ensure that its recommendations regarding these problems are implemented.	5.00	1.60	5.55	1.26
Policymakers should consequently implement the recommendations from science to resolve these problems.	5.75	1.18	5.52	1.18
Science should clearly tell policymakers how to address these problems.	5.57	1.44	4.76	1.69
In case of doubt, science should be able to intervene in policy regarding these problems.	5.30	1.52	3.74	1.89
*We should. . .*	Political ideology			
	*M*	*SD*	*M*	*SD*
. . .strive for a society that is as diverse as possible.	6.04	1.22	5.57	1.41
. . .pronounce stricter prison sentences for criminals.	5.17	1.60	4.84	1.70
. . .accept fewer refugees.	3.14	1.52	3.17	1.43
. . .use private-sector profits to finance schools and other public services.	5.18	1.78	5.23	1.62
. . .reduce income inequality in society through new taxes.	5.50	1.65	5.19	1.73
. . .increase support for the unemployed.	5.57	1.36	4.74	1.52

The table displays mean answers of 205 to 204 U.S. and 173 to 168 German scientists to the survey items; participants with missing answers were excluded. Items were rated on a 7-point Likert-type scale (1 = do not agree at all; 7 = absolutely agree). In the case of self-images, the first three items capture political involvement, the next three scientific neutrality, and the final three epistemic authority.

#### Wish for science to direct policy-making

Following Post et al. (2021), I measured respondents’ views on the role of science in policy-making with a modified scale tailored to the research context capturing participants’ wish for science to direct policy-making (cf. Table 1). I asked participants, “How should society ideally handle problems that touch on scientific issues, such as the COVID-19 pandemic?” Then, participants rated four different statements on a 7-point Likert-type scale (1 = do not agree at all; 7 = absolutely agree) capturing the belief that science should direct policy-making (“ensuring implementation of recommendations” or “telling policymakers how to address problems”). A PCA revealed that four items load on one factor in both samples, accounting for 55% of the variance (χ^2^(6) = 107.52; *df* = 6; *p* < .001; *KMO* = .71; Supplemental Material 3 for factor loadings). I averaged respondents’ ratings (*Cronbach’s α* = .74). ^5^

#### Importance to communicate policy advice

Policy advice means assessing how proposed actions affect societal priorities. During the time of the COVID-19 pandemic covered in this study, this included communicating whether the situation was to be considered a pandemic or endemic to the public. I devised a set of items to measure the importance pandemic scientists attributed to communicating such policy advice (cf. Table 2; some items adapted from Post and Ramirez, 2018). Respondents were asked, “What should be considered when communicating the study results to the public?” Then, they rated various considerations on a 7-point Likert-type scale (1 = very unimportant; 7 = very important). Five items were chosen to capture the communication of policy advice drawing attention to study results and their implications for policy disputes (“initiating a debate” or “clarifying political relevance”). Through a PCA, I examined how these items group together, indicating a single underlying dimension, accounting for 47% of the variance (χ^2^(10) = 153.97; *p* < .001; *KMO* = .76; Supplemental Material 3 for factor loadings). Thus, I averaged respondents’ ratings (*Cronbach’s α* = .71).

**Table 2.** Survey items, importance to communicate policy advice across scenarios.

*How important is it to. . .*	U.S. scientists					
	Endemic		Pandemic		Control	
	*M*	*SD*	*M*	*SD*	*M*	*SD*
. . .initiate a debate on political measures?	3.76	1.56	3.81	1.63	3.95	1.63
. . .draw attention to this important topic?	5.82	1.18	5.44	1.30	6.06	1.21
. . .warn society against wrong decisions?	5.55	1.52	5.15	1.67	5.65	1.28
. . .provide decision support for politics?	5.32	1.58	5.24	1.49	5.52	1.50
. . .clarify the political relevance of the results?	4.86	1.68	4.88	1.76	4.77	1.76
*How important is it to. . .*	German scientists					
	*M*	*SD*	*M*	*SD*	*M*	SD
. . .initiate a debate on political measures?	4.43	1.53	4.52	1.53	4.47	1.37
. . .draw attention to this important topic?	5.59	1.17	5.40	1.31	5.66	1.02
. . .warn society against wrong decisions?	4.96	1.71	5.46	1.25	5.53	1.30
. . .provide decision support for politics?	5.49	1.26	5.49	1.29	5.64	1.07
. . .clarify the political relevance of the results?	4.98	1.52	6.11	1.65	5.02	1.25

The table displays mean answers of 204 U.S. and 174 German scientists to the survey items in each scenario of the survey experiment. Items were rated on a 7-point Likert-type scale (1 = very unimportant; 7 = very important).

#### Control

Apart from the conceptual variables, I controlled for pandemic scientists’ gender (1 = female), seniority level (1 = professors, research group leaders, assistant professors, and junior research group leaders), and stance regarding the validity of containment measures (“Given the already sustained spread of SARS-CoV-2, further containment measures are disproportionate at this time,” 1 = permissible; 5 = inadmissible). I also controlled for political ideology using a six-item scale (adapted from Hooghe et al., 2002) loading on a single factor (χ^2^(10) = 316.70; *p* < .001; *KMO* = .764; *Cronbach’s α* = .71; cf. Table 1 and Supplemental Material 3 for factor loadings), encompassing both cultural and socioeconomic dimensions of political ideology (1 = conservative; 7 = progressive).

## Results

### Self-images

I asked how pandemic scientists conceive of themselves in policy disputes (RQ1). To map pandemic scientists’ self-images, I surveyed them for their political involvement, stance on neutrality, and sense of epistemic authority. However, previous research suggests that scientists’ self-images are often complex and not neatly separated, so overlap between the previously identified dimensions is to be expected. To address these difficulties, I conducted a latent profile analysis (LPA) using the software package *tidyLPA* in R (Rosenberg et al., 2018). The method allows for overlaps and segments participants into groups with similar self-images according to their responses to the nine indicator items capturing political involvement, stance on neutrality, and epistemic authority.

Following guidelines from Collins and Lanza (2009), the approach involved multiple steps. I first eliminated seven outliers whose responses on the indicator variables deviated more than three standard deviations from the mean. To account for non-normal data distribution, I employed the Maximum-Likelihood Estimator with robust error calculation. I contrasted model specifications with different numbers of groups to evaluate measures like the Akaike information criterion (AIC) and Bayesian information criterion (BIC), as lower values in these parameters are indicative of a better model fit. Entropy was used to gauge the clarity of group assignments, with higher values indicating distinct classifications. Finally, I examined the probabilities of individual-to-group assignments and their group sizes (cf. Supplemental Material 4 and 5). As a rule of thumb, the average probability that respondents belong to an assigned class and no other should be greater than .70 and no profile should contain less than 5% of the respondents (cf. Stanley et al., 2017). In the end, I chose a model with four groups of pandemic scientists. I assessed the findings’ robustness through validations (cf. Supplemental Material 4).

**Figure 1.**
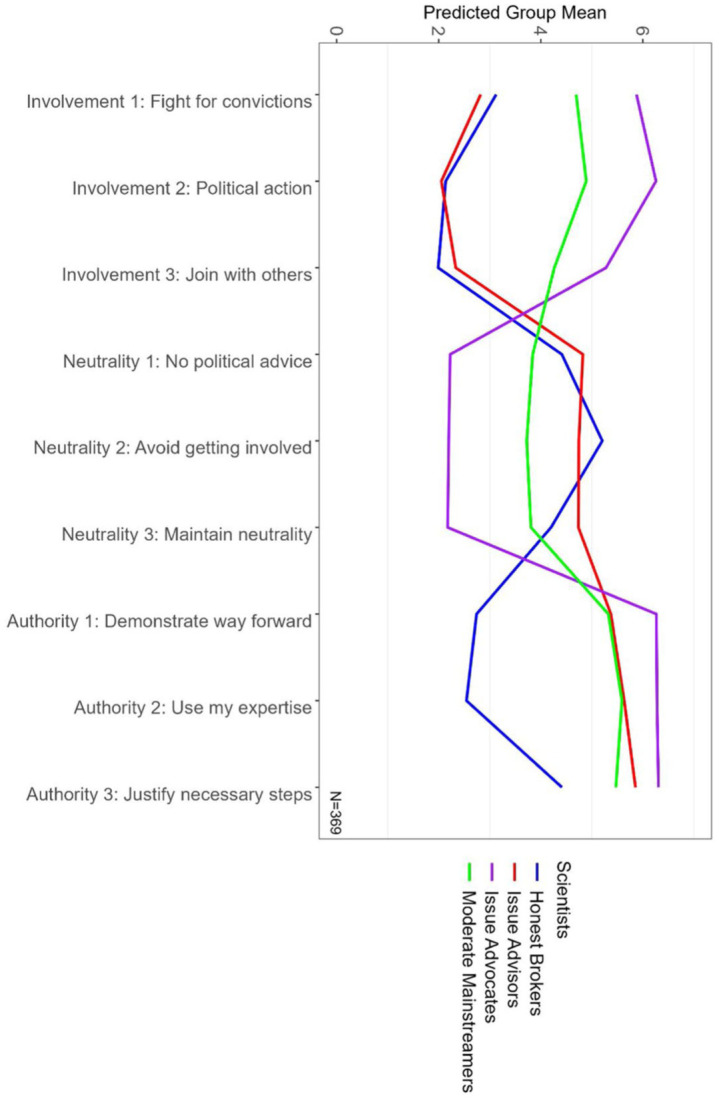
Predicted mean responses to indicator variables (capturing self-images). The figure illustrates the predicted mean responses (1 = do not agree at all; 7 = absolutely agree) to the indicator items (cf. chapter self-images) of the four identified groups of scientists; participants with missing answers were excluded from the analysis. The four groups represent varying degrees of political involvment, scientific authority, and epistemic authority. The wordings of the indicator variables are displayed in Table 1. The indicator items appeared in the same order as listed in Table 1, with Involvement 1 being “In societal debates, I fight for my political convictions.” Note: for color figure please see online version.

The identified four-group model^6^ shows an entropy score of *H* *=* .83, indicating clear classification. The probabilities of individual-to-group assignments range from *P_min_* = .82 to *P_max_* = .93 and demonstrate strong classification reliability (cf. Figure 1). Moreover, post hoc analyses from one-way ANOVAs show that the identified groups differ in their average ratings across the three dimensions I surveyed pandemic scientists on (cf. Supplemental Material 3, Table 3 for details), namely the scales political involvement (*F*(3) = 350.35, *p* < .001), scientific neutrality (*F*(3) = 71.47, *p* < .001), and epistemic authority (*F*(3) = 145.80, *p* < .001).

**Table 3.** Multinomial regressions explaining group membership.

	U.S. scientists			German scientists		
	Advisers	Advo.	Broker	Advisers	Advo.	Broker
	*Exp(B)*	*Exp(B)*	*Exp(B)*	*Exp(B)*	*Exp(B)*	*Exp(B)*
Wish for science to direct policy-making (1: low; 7: high)	.98	2.02**	1.27	.86	.88	.96
Stance on containment measures (1: permissible; 5: inadmissible)	1.23	.87	.82	1.19	1.49	1.73*
Political ideology (1: conservative; 7: progressive)	.70	2.28**	.34***	.79	2.70*	.88
Seniority (1: professorship)	1.82	2.24	.69	2.36*	.95	.37
Gender (1: female)	1.09	1.20	3.12	3.22**	.87	2.77*
*Nagelkerke R^2^*	.272***			.239***		

The table shows multinomial regression results predicting group membership (Issue Advisors, Issue Advocates, and Honest Brokers, with Moderate Mainstreamers as the reference category) among 190 U.S. and 159 German scientists; participants with missing answers or classifications were excluded from the analysis.

Significance levels are marked as ***p < .001, **p < .01, *p < .05.

The largest group (45%) exhibits moderate responses and is termed *Moderate Mainstreamers*. These pandemic scientists see themselves as moderately involved in politics (*M* = 4.61, *SD* = 0.78), while expressing only limited commitment to scientific neutrality (*M* = 3.78, *SD* = 1.14). At the same time, they tend to claim some epistemic authority for themselves (*M* = 5.46, *SD* = 0.83), suggesting they see their expertise as relevant in policy disputes while expressing ambivalence about their role in policy disputes. Moderate Mainstreamers thus stand apart in their ambivalence.

The second-largest group (29%) emphasizes scientific neutrality and epistemic authority, and is termed *Issue Advisors*. These pandemic scientists see themselves as detached from politics (*M* = 2.37, *SD* = 0.73) and committed to scientific neutrality (*M* = 4.81, *SD* = 1.22). They nonetheless claim epistemic authority (*M* = 5.62, *SD* = 0.77), suggesting they see their expertise as relevant in policy disputes while not considering themselves political actors. Issue Advisors stand apart by claiming to be detached from politics while still embracing a certain epistemic authority over policy disputes.

The third group (14%), termed *Issue Advocates* in line with Pielke’s typology, stands out as the only group that does not distance itself from politics. These pandemic scientists see themselves as highly politically involved (*M* = 5.87, *SD* = 0.73) and largely unconcerned with maintaining scientific neutrality (*M* = 2.06, *SD* = 0.95). They also claim the highest epistemic authority (*M* = 6.33, *SD* = 0.55), suggesting they understand themselves as involved citizens whose expertise is relevant in policy disputes. Issue Advocates stand apart as they are the only pandemic scientists potentially blurring boundaries between science and politics.

The smallest group (12%) is termed Honest Brokers, in line with Pielke’s typology, as they neither claim epistemic authority nor engage in politics. These pandemic scientists see themselves as detached from politics (*M* = 2.38, *SD* = 0.93) and committed to scientific neutrality (*M* = 4.67, *SD* = 1.41). At the same time, they claim the lowest epistemic authority (*M* = 3.21, *SD* = 0.77). Honest Brokers stand apart as they are the only pandemic scientists who do not assert epistemic authority over policy disputes. Therefore, it is plausible that these pandemic scientists are the only ones who see themselves as public experts in an ideal sense.

Altogether, the Latent Profile Analysis reveals distinct self-images among the surveyed pandemic scientists. While two groups closely align with Pielke’s typology of public experts, the empirical analysis indicates that Moderate Mainstreamers and Issue Advisors reflect a more nuanced position regarding their integration into policy disputes.

### Wish for science to direct policy-making

On average, U.S. (*M* = 5.40, *SD* = 1.16) and German pandemic scientists surveyed (*M* = 4.88, *SD* = 1.08) express a clear preference for a strong role of science in policy-making. Only 10% of U.S. and 12% of German scientists surveyed are skeptical with mean scores below the midpoint of the four-item scale. However, U.S. scientists show a notably higher tendency to endorse science to direct policy-making (*t*(377) = −4.53, *p* < .001, Cohen’s *D* = 1.12). The results demonstrate a near consensus among participants on the pivotal role of science in policy disputes.

I asked how pandemic scientists’ self-images are associated with their wish for science to direct policy-making (RQ2). Thus, I am interested in whether the identified groups differ in their ratings, and particularly whether Issue Advocates express a stronger wish for science to direct policy-making (as these scientists potentially blur the boundaries between science and politics). Exploring this assumption, I utilized multinomial logistic regression. I estimated group membership (to the Issue Advisors, Issue Advocates, and Honest Brokers) by respondents’ approval of science dominating policy-making. The largest group, the Moderate Mainstreamers, served as a reference category. Multinomial logistic regression was chosen due to the categorical nature of group membership, allowing for the consideration of non-linear relationships between variables. I controlled for stance on containment measures, political ideology, seniority, and gender.

Table 3 suggests that pandemic scientists’ wish for science to direct policy-making, stance on containment measures, political ideology, seniority, and gender are related to self-images that they hold. U.S. pandemic scientists, who highly wish for science to direct policy-making, tend to adopt a more active approach to policy disputes as Issue Advocates (*Exp(B)* = 2.02, *p* = .003). Notably, pandemic scientists with a progressive political ideology are more likely to identify as Issue Advocates, a pattern observed in both U.S. (*Exp(B)* = 2.28, *p* = .005) and German samples (*Exp(B)* = 2.70, *p* = .011).

In summary, the findings emphasize an association between pandemic scientists’ wish for science to direct policy-making and their self-perceptions, particularly in the U.S. context. Here, the assumption underlying RQ2 appears to hold true: Issue Advocates wish for a greater dominance of science in policy-making. The findings suggest that the U.S. pandemic scientists surveyed, especially Issue Advocates, place higher importance on science in policy-making compared with their German colleagues, likely due to the more polarized political environment in the United States (RQ3).

### Selective communication of policy advice

To test the final two hypotheses—whether the pandemic scientists are willing to communicate policy advice only in accordance with their own policy preferences (H1), and whether Issue Advocates, who potentially blur the boundaries between science and politics, are particularly willing to do so (H2)—I conducted a between-subject experiment. I presented respondents either with a study outcome, suggesting an endemic or pandemic situation, or a control condition. Afterwards, I measured how much importance they attributed to communicating policy advice related to the study outcomes.

The U.S. pandemic scientists surveyed show an affinity for communicating policy advice when presented with research results indicating an endemic status of COVID-19 (*M* = 5.06, *SD* = 1.06), a pandemic status (*M* = 4.90, *SD* = 1.14), and in the control condition (*M* = 5.19, *SD* = 0.91). The German pandemic scientists surveyed show a similar tendency when presented with results indicating an endemic status of COVID-19 (*M* = 5.09, *SD* = 0.96), a pandemic status (*M* = 5.20, *SD* = 1.03), and in the control condition (*M* = 5.26, *SD* = 0.80). Responses do not vary by experimental materials or national context (*F*(5) = 1.08, *p* = 0.369).

I conducted a multivariate analysis of variance (ANOVA) to test the first and second hypothesis, examining the importance respondents place on communicating policy advice across experimental materials (cf. Table 4). I created two groups of pandemic scientists based on opposing stances on containment measures: one group viewing containment measures as permissible or mostly permissible (93 U.S. and 58 German pandemic scientists), and the other comprising those who viewed them as inadmissible, largely inadmissible, or uncertain (111 U.S. and 116 German pandemic scientists). If pandemic scientists are inclined to align their policy advice with their personal policy preferences, I would see an interaction effect between the materials and their stance on containment measures. Then pandemic scientists might, for example, hesitate to highlight policy advice that contradicts their stance. Moreover, if the second hypothesis on Issue Advocates holds true, I would observe a three-way interaction effect among materials, stance on containment measures, and self-images.

**Table 4.** Multivariate analysis of covariance explaining importance of policy advice.

	U.S. scientists			German scientists		
	*df*	*F*	*p*	*df*	*F*	*p*
Materials	2	.08	.775	2	1.57	.212
Stance on containment measures	1	.05	.821	1	2.95	.088
Self-images	3	25.55	<.001	3	11.72	<.001
Self-images × stance	3	4.64	.032	3	4.98	.027
Materials × stance	2	.59	.443	2	.12	.725
Materials × self-images	6	.17	.682	6	.50	.479
Materials × stance × self-images	5	.04	.833	5	.733	.393

The table shows the results of a multivariate analysis of variance results predicting affinity toward communicating policy advice related to study outcomes presented in the experimental materials (scenarios) among 190 U.S. and 159 German scientists; participants with missing answers or classifications were excluded from the analysis. Equal variance according to Levene’s Test of Equality of Error Variances and normally distributed dependent variables across groups can be assumed.

However, the analysis reveals no substantial interaction between the experimental materials and the stances of pandemic scientists on containment measures. Moreover, there is no interaction effect between all three factors. However, Issue Advocates consistently emphasize the relevance of study results more strongly, irrespective of the findings (cf. Supplemental Material 6). These results underscore that, contrary to my expectations, the pandemic scientists surveyed emphasize the importance of communicating policy advice, regardless of their own stances (H1 and H2). Furthermore, there are no differences in this regard between U.S. and German scientists (RQ3).

## Discussion

The study contributes to the field of science communication by examining how pandemic scientists’ conceive of themselves in policy disputes (cf. RQ1). The identification of four distinct groups—Moderate Mainstreamers, Issue Advisors, Issue Advocates, and Honest Brokers—provides a framework to examine scientists’ engagement in policy disputes. The classification echoes some aspects of Pielke’s (2007) typology of scientific expertise, but avoids portraying scientists as stereotypical figures and allows for overlap and contradiction. Notably, the Moderate Mainstreamers and Issue Advisors, representing the largest cohorts, demonstrate a nuanced role that does not align with Pielke’s categories. Scientists may see themselves as neutral even while justifying intervening in policy disputes based on their superior expertise.

The differences in the wish for science to direct policy-making further underline the varying approaches to policy disputes among pandemic scientists (RQ2). Pielke (2004) suggested that the value of science in policy-making is overestimated especially by scientists who blur the boundaries between science and policy-making, an assumption that the study found to hold true only for U.S. respondents. The overall higher tendency of U.S. pandemic scientists to endorse the dominance of science in policy-making, compared with their German counterparts, may reflect cultural or systemic differences in the integration of science into policy-making (RQ3). This divergence prompts further exploration into how national contexts shape scientists’ views on their role in policy disputes.

I did not find any evidence of pandemic scientists selectively communicating policy advice (H1 and H2). I therefore assume that the accusation of stealth advocacy, for example, can only be applied to minorities of scientists on the fringes. This warrants further examination in future research. However, I dispel an accusation that remains a common narrative post-COVID-19: the pandemic scientists surveyed show no interest in distorting policy disputes.

The analysis also reveals a noteworthy connection between the self-images scientists hold and their contributions to policy disputes. Most notably, Issue Advocates in the United States and Germany display the highest willingness to communicate policy advice. This suggests a potential alignment between national context and scientists’ professional conduct and ethos (RQ3).

The research findings align closely with Post and Bienzeisler (2024), who examined how scientists’ engaging as Honest Brokers or Epistocrats influence public trust in science in policy disputes. They found that Honest Brokers, who clearly separate scientific facts from policy advice, help reduce trust polarization in politically sensitive issues compared with Epistocrats who blur the lines between scientific and political claims. This complements the discovery of different scientist groups and their varying approaches. Specifically, Issue Advocates blur scientific and political lines visibly, potentially leading to divided public trust. This highlights the importance of scientists’ communication and engagement in policy disputes on public trust, especially among politically sensitive audiences.

Certain limitations should be considered when interpreting the results. First, as a predominantly correlational study, it limits causal inferences. The relationships observed between pandemic scientists’ responses and their assessments of communication behaviors, while suggestive, cannot definitively establish causality. Second, the study’s design, based on self-reported survey data, raises questions about how expressed attitudes reflect actual behavior. Third, the generalizability of the findings is limited due to the sampling approach and the unique conditions of the COVID-19 pandemic. Primarily consisting of U.S. and German medical scientists, the convenience sample may not fully represent the broader scientific community across other fields or regions. In addition, the reliance on voluntary participation, without incentives, and a low recruitment rate could have introduced biases. For example, one might assume that the participants were primarily those already engaged in policy disputes. However, I do not find evidence for this. Self-reported data from other parts of the survey indicate that majority of respondents are, in fact, not overly publicly engaged: for example, only 38% of U.S. and 21% of German respondents had issued a position statement in the previous year, and just 65% of U.S. and 52% of German respondents had spoken to a newspaper. Given the circumstances of a global pandemic, these levels of engagement are relatively low. Thus, I believe my sampling approach is valid, although I cannot conclusively demonstrate its validity.

Future studies should aim to employ more sophisticated experimental designs for causal analysis and broaden the sample to encompass a wider range of scientific disciplines and geographic areas.

## Conclusion

Almost a century ago, Merton (1945: 414) wrote that the “honeymoon of intellectuals and policymakers is often nasty, brutish, and short.” My research indicates that some scientists may overestimate the role science plays in policy-making. In my eyes, scientists, citizens, and policymakers have misconceptions about the nature of each other’s contributions to functioning democracies. Debates over complex issues involve both values and scientific evidence. In practice, this can be one of the reasons why some scientists think they live in a post-truth society (cf. Iyengar and Massey, 2019).

The findings add to the increasing series of studies empirically testing scientists’ beliefs and attitudes. This includes studies looking at their communicative objectives (Alinejad and van Dijck, 2022; Banse et al., 2025; Besley and Nisbet, 2013; Graminius, 2023), mental models of science communication (Kessler et al., 2022), or normative orientations (Bray and von Storch, 2017). This line of research shows that scientists should not be conceived as objective observers, but as individuals shaped by ideologies, values, and biases. So far, this point has often been ignored—both empirically and theoretically. I hope further research will address this gap by examining additional beliefs and attitudes.

## Supplemental Material

sj-pdf-1-pus-10.1177_09636625251371565 – Supplemental material for Of Issue Advocates and Honest Brokers: Participation of U.S. and German scientists in COVID-19 policy disputes Supplemental material, sj-pdf-1-pus-10.1177_09636625251371565 for Of Issue Advocates and Honest Brokers: Participation of U.S. and German scientists in COVID-19 policy disputes by Nils Bienzeisler in Public Understanding of Science

## Notes

1. The literature uses several terminologies to describe collective identities. Some approaches focus on professional cohesion (e.g. Banse et al., 2025; Fischer and Schmid-Petri, 2023), while others discuss collective political identities (e.g. Dvir-Gvirsman, 2019; Tajfel and Turner, 2004). All these approaches assume that communities are built on shared ideas of who they are. Since scientists can be involved in policy disputes both as professionals and as citizens, I refer to collective identities that encompass both aspects.

2. The response rates in this study are comparable with those in similar surveys (e.g. Besley et al., 2018; Bray and von Storch, 2017). I assume that the lower response rate in the United States may be due to the sampling approach, where participants were contacted via unsolicited emails containing a direct survey link; it is highly possible that some of these emails were marked as spam (which, from the perspective of IT departments, would be desirable).

3. Due to considerations of data protection, age was surveyed in ranges of 5 years.

4. Among U.S. scientists, the experimental groups were comparable in terms of gender distribution (χ^2^(2) = 3.62, *p* = .164), and seniority (χ^2^(2) = 3.01, *p* = .222). Among German scientists, the experimental groups were comparable in terms of gender distribution, although the control condition had slightly more females (χ^2^(2) = 6.35, *p* = .042), while no difference was found regarding seniority across conditions (χ^2^(2) = 3.29, *p* = .193). Pre-existing attitudes toward containment measures were distributed equally across the conditions in both the United States (*F*(2) = 1.764, *p* = .174) and Germany (*F*(2) = 1.829, *p* = .164). Political ideology was also equally distributed across conditions in both Germany (*F*(2) = 0.436, *p* = .647) and the United States (*F*(2) = 0.790, *p* = .455).

5. The items proved to be just suitable for forming a mean score in the subpopulations, with *Cronbach’s α* = .81 for the United States and *Cronbach’s α* = .64 for German scientists. This suitability is supported by the exploratory nature of the study and the scale, making slightly lower reliability values still acceptable (cf. Cortina, 1993).

6. I initially conducted the LPA separately for U.S. and German researchers and found the results to be remarkably similar between the two samples. This close alignment in findings across distinct national contexts underscores the robustness of the model. Consequently, I decided to merge both samples for a more comprehensive model specification.
