# Supplementary material for: Of Issue Advocates and Honest Brokers: Participation of U.S. and German scientists in COVID-19 policy disputes
Source: Public Underst Sci. 2025 Oct 17;35(2):177–96. doi: 10.1177/09636625251371565 (PMC12852491; doi:10.1177/09636625251371565)
Supplement: sj-pdf-1-pus-10.1177_09636625251371565 – Supplemental material for Of Issue Advocates and Honest Brokers: Participation of U.S. and German scientists in COVID-19 policy disputes [file sj-pdf-1-pus-10.1177_09636625251371565.pdf]

Supplemental Materials

Of Issue Advocates and Honest Brokers

Participation of U.S. and German Scientists in COVID-19 Policy Disputes

Nils Bienzeisler

Table of Contents

Supplemental Material 1: Sample	p. 1
Supplemental Material 2: Overview of experimental materials	p. 2
Supplemental Material 3: Scales	p. 3
Supplemental Material 4: Validation of Latent Profile Analysis	p. 7
Supplemental Material 5: Details on Latent Profile Analysis	p. 10
Supplemental Material 6: Descriptive results survey experiment	p. 12

Supplemental Material 1: Sample

Table 1: Sample

	U.S. scientists		German scientists	
Gender	*n*	%	*n*	%
Female	85	41.5	76	43.7
Male	120	58.5	98	56.3
Age	*n*	%	*n*	%
18-24 years	0	0	0	0
25-29 years	7	3.4	9	5.2
30-34 years	17	8.3	21	12.1
35-39 years	20	9.8	18	10.3
40-44 years	22	10.7	31	17.8
45-49 years	26	12.7	23	13.2
50-54 years	22	10.7	13	7.5
55-59 years	30	14.6	20	11.5
60-64 years	21	10.2	26	14.9
65+ years	36	17.6	8	4.6
Professional status	*n*	%	*n*	%
Doctoral student	9	4.4	18	10.3
Post-doctoral researchers	11	5.4	70	40.2
Assistant professors	37	18.0	10	5.7
Professor	138	67.3	64	36.8
Others	10	4.9	12	6.9
Disciplinary background	*n*	%	*n*	%
Biology	14	6.8	20	11.7
Epidemiology	18	8.8	20	11.7
Immunology	19	9.3	11	6.4
Computer science/statistics	9	4.4	17	9.9
Internal Medicine	7	3.4	9	5.3
Psychiatry and Psychology	22	10.7	25	14.6
Public Health	63	30.7	26	15.2
Virology	16	7.9	24	13.5
Other	37	18.0	18	10.3
	M	*SD*	*M*	*SD*
Political ideology (1: conservative; 7: progressive)	5.55	1.01	5.41	1.00

Notes: The table shows the distribution of respondents in the U.S. and German samples by age, gender, political ideology, professional status, and disciplinary background. Information on “other” professional status and disciplinary background can be found in the dataset uploaded to OSF.

Supplemental Material 2: Overview of experimental materials

Table 1: Experimental materials

Experimental materials		
Condition: Epidemic	Condition: Pandemic	Control
Suppose colleagues have conducted a research project on SARS-CoV-2. The results have been published in a prestigious journal. One conclusion that can be drawn from the results is that SARS-CoV-2 is widespread and there is a corresponding immunity in the population, so that one can speak of an endemic spread.	Suppose colleagues have conducted a research project on SARS-CoV-2. The results were published in a prestigious journal. One conclusion that can be drawn from the results is that although SARS-CoV-2 is widespread and there is immunity in the population, it is still *not* endemic.	Suppose colleagues have conducted a research project on SARS-CoV-2. The results were published in a prestigious journal.

Note: The table displays the experimental materials used in this study. I presented each participant with one of three scenarios about a research project on SARS-CoV-2. The materials were varied to evaluate pandemic scientists' views on the appropriateness of using different scientific findings for policy advice

Supplemental Material 3: Scales

Table 1: Self-images scale

	Political involvement	Scientific neutrality	Epistemic authority
In societal debates, I...	λ	λ	λ
...fight for my political convictions.	.72	–.35	
...take political action for social change.	.82	–.26	.21
...join with others to build majorities for meaningful political action.	.84		.20
...take care not to give political advice.		.79	
...avoid getting involved in political conflicts whenever possible.		.82	
...maintain neutrality and independence from conflicting parties.		.74	
...strive to demonstrate to politics and society the best way forward with my scientific findings.	.21		.77
...use my expertise to work toward the implementation of scientifically appropriate measures.	.25		.80
...use my knowledge as an expert to justify necessary steps.			.81

Note: The table displays the results of a PCA with varimax rotation examining items answered by the respondents to the question "*How do you personally contribute to societal debates in your field?*" to access their self-images (1: do not agree at all; 7: absolutely agree). Only factor loadings $\lambda \geq .2$ are displayed. The PCA revealed that each three items load on one of three factors, accounting together for 68.0% of the variance ($\chi 2$ (36) = 1040.40; $p < .001$; *KMO* = .77). The results of the PCA are not presented in the main document as the items derived from the PCA were subsequently utilized in the LPA models. However, the answers of the respondents stating their *involvement in politics* (*Cronbach's α* = .79), *stance on neutrality* (*Cronbach's α* = .73), and *epistemic authority* (*Cronbach's α* = .74) can be used to form composite scores.

Table 2: Correlation matrix self-images scale

Item	1	2	3	4	5	6	7	8	9
1	1.00	.58	.45	-.35	-.36	-.34	.19	.21	.16
2	.58	1.00	.62	-.30	-.38	-.32	.33	.34	.19
3	.45	.62	1.00	-.22	-.24	-.13	.30	.32	.21
4	-.35	-.30	-.22	1.00	.56	.39	-.12	.03	-.05
5	-.36	-.38	-.24	.56	1.00	.47	-.13	-.15	-.17
6	-.34	-.32	-.13	.39	.47	1.00	-.03	-.04	.02
7	.19	.33	.30	-.12	-.13	-.03	1.00	.55	.43
8	.21	.34	.32	.03	-.15	-.04	.55	1.00	.49
9	.16	.19	.21	-.05	-.17	.02	.43	.49	1.00

Note: The table displays a correlation matrix of the nine items answered by the respondents to the question "*How do you personally contribute to societal debates in your field?*" to access their self-images (1: do not agree at all; 7: absolutely agree). Numbers indicate survey items, displayed in the same order as in Table 1 with 1 being the item, *"In societal debates, I fight for my political convictions."* and 9, *"In societal debates, I use my knowledge as an expert to justify necessary steps."*

Table 3: Wish for science to dominate policy-making scale

	λ
Science should ensure that its recommendations regarding these problems are implemented.	.63
Policymakers should consequently implement the recommendations from science to resolve these problems.	.77
Science should clearly tell policymakers how to address these problems.	.77
In case of doubt, science should be able to intervene in policy regarding these problems.	.78

Note: The table displays the results of a PCA examining items answered by the respondents to the question *"How should society ideally handle problems that touch on scientific issues, such as the COVID-19 pandemic?"* to access their wish for science to dominate policy-making (1: do not agree at all; 7: absolutely agree). The PCA revealed that the items load on one factor, accounting for 56.1% of the variance ($\chi 2$ (6) = 107.52; $df = 6$; $p < .001$; *KMO* = .71). I averaged respondents' ratings (U.S. pandemic scientists: *Cronbach's* α = .82; German pandemic scientists *Cronbach's* α = .64).

Table 4: Importance to communicate policy advice scale

How important is it to...	λ
...initiate a debate on political measures?	.67
...draw attention to this important topic?	.67
...warn society against wrong decisions?	.68
...provide decision support for politics?	.74
...clarify the political relevance of the results?	.66

Note: The table displays the results of a PCA examining items answered by the respondents to access their willingness to communicate policy advice (1: very unimportant; 7: very important), as presented in my survey experiment and factor loadings λ. The PCA revealed that the items load on one factor, accounting for 46.8% of the variance ($\chi 2$ (10) = 153.; $p < .001$; *KMO* = .76). I averaged respondents' ratings (U.S. pandemic scientists: *Cronbach's* α = .72; German pandemic scientists *Cronbach's* α = .72).

Table 5: Political ideology scale

We should…	λ
...strive for a society that is as diverse as possible.	.57
...pronounce stricter prison sentences for criminals.	.71
...accept fewer refugees.	.38
...use private-sector profits to finance schools and other public services.	.72
...reduce income inequality in society through new taxes.	.72
...increase support for the unemployed.	.737

Note: The table displays the results of a PCA examining items answered by the respondents to access their political ideology (1: do not agree at all; 7: absolutely agree) encompassing both cultural and socio-economic dimensions of political ideology (adapted from Hooghe et al., 2002). The PCA revealed that the items load on one factor *only*, accounting for 42.7% of the variance ($\chi 2$ (15) = 188.81; $p < .001$; *KMO* = .75). I averaged respondents' ratings (U.S. pandemic scientists: *Cronbach's α* = .75; German pandemic scientists *Cronbach's α* = .68).

Supplemental Material 4: Validation of Latent Profile Analysis

The validation of Latent Profile Analysis (LPA) is crucial, as it is a data-driven approach. Results must be theoretically plausible and robust. First, we examined whether German and U.S. researchers responded similarly to the scales. Second, the model was checked for internal validity, examining if latent groups represent distinct patterns. Third, stability was verified through sensitivity analyses. Fourth, construct validity was assessed by contextualizing the results within theory. Fifth, external validity was evaluated focusing on the applicability of the findings to other contexts or populations.

First, to examine whether U.S. and German scientist differ in their responses to the survey items, I conducted a measurement invariance analysis. This method tests whether a latent construct is measured in the same way across different groups. Using confirmatory factor analysis, the procedure checks whether factor structures and loadings are sufficiently similar. Three increasingly restrictive models are compared. The configural invariance model tests whether the same items load on the same factors across groups, allowing parameters to vary. The metric invariance model additionally tests whether the strength of these loadings is comparable, holding them constant across groups. Finally, the scalar invariance model tests whether mean differences in item responses exist, holding intercepts constant across groups. Measurement invariance analysis showed acceptable fit for the configural model (*CFI* = .943, *RMSEA* = .081, *SRMR* =.054), indicating that the three-factor structure is comparable across German and U.S. pandemic scientists. Metric ($\Delta\chi^2(6)$ = 13.65, p = .034) and scalar invariance ($\Delta\chi^2(6)$ = 12.85, p = .045) showed small effects. This suggests only minor differences in how some items are interpreted

Second, I checked if the profiling dimensions–political involvement, scientific neutrality, and epistemic authority—differ among groups (cf. Appendix 5, Table 3). I used one-way ANOVAs, which confirmed differences for scores created using the items for political

involvement ($F(3) = 350.35$, $p < .001$), scientific neutrality ($F(3) = 71.47$, $p < .001$), and epistemic authority ($F(3) = 145.80$, $p < .001$). Additionally, I considered a five-class solution, but this model did not provide as clear a classification compared to the four-class model (cf. Figure 1).

Figure 1: Comparing predicted mean responses of identified groups across samples.

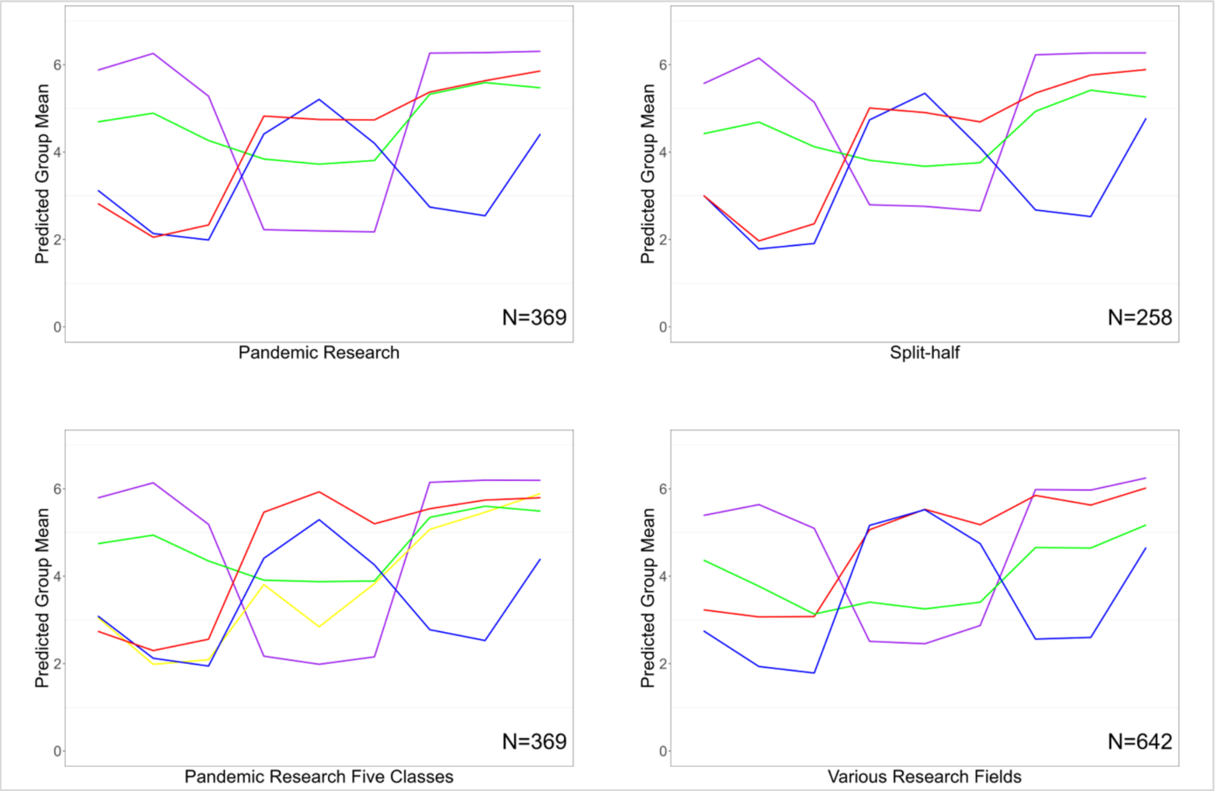

Note: The figure illustrates the predicted mean responses (1: do not agree at all; 7: absolutely agree) of groups of scientists across different samples and with different model specifications. The first graph in the series illustrates the model employed, the second graph utilizes a training dataset comprising only 70% of the responses to demonstrate model robustness, the third graph explores the impact of adding a fifth class (highlighted in yellow), and the final graph displays the distribution of responses of scientists across various research topics (biodiversity, linguistics, COVID-19). The indicator items appeared in the same order as listed in Table 1, with the first point being *"In societal debates, I fight for my political convictions."*

Third, we tested the robustness of the model. Separate analyses for different research fields and cross-validation with a training dataset (70%) were conducted, each supporting a four-group model (cf. Figure 1). Group-specific mean distributions were comparable to the overall model, indicating robust model structure, although minor differences were noted, possibly due to sample variance or dataset characteristics. I also included the covariates gender

and seniority in an additional model and yielded similar results. Thus, both sensitivity analyses yielded comparable results, suggesting sufficient model robustness.

Fourth, to assess construct validity, I examined whether group memberships align with theoretical expectations. I analyzed differences in perceptions of scientific tasks, using scales for the importance of generating scientific knowledge and political action. ANOVA results showed group differences in perceptions of science's role in political action ($F(3) = 14.61$, $p < .001$), but not in research orientation ($F(3) = 2.07$, $p = .104$), supporting a plausible theoretical connection.

In addition to the quantitative validation, I conducted 12 follow-up qualitative interviews with German pandemic scientists. The interviews were coded to align with the survey dimensions of the scope of expertise scale. I matched the interviewees with their corresponding class assignments derived from the quantitative survey. The subsequent qualitative analysis corroborated the group characteristics identified in the LPA, with one example being the Issue Advocates, whose members were more often engaged in political actions and more likely to utilize their authority for advocacy than others, aligning with their profile in the quantitative analysis. The interviewed members of the Issue Advocates also perceived activism as a viable option for enacting change.

Fifth, the external validity of the LPA was considered. The survey data's opportunistic sample may limit generalizability, but demographic data suggests diverse representation. I further tested the scale with both linguistic and biodiversity researchers, finding that the results were largely consistent with those obtained from the initial grouping, reaffirming the applicability across diverse scientific disciplines (cf. Figure 1). Moreover, theoretical literature supports the results, indicating potential applicability beyond the studied contexts, though the smallest group's generalizability remains uncertain.

Supplemental Material 5: Details on Latent Profile Analysis

Table 1: Model specifications LPA

Classes of Scientists	*AIC*	*BIC*	*Entropy*	$Prob_{min}$	$Prob_{max}$	*BLRT p-Value*
1	12875.45	12945.84	1.0	1.0	1.0	
2	12336.49	12445.99	.85	.95	.97	>.001
3	12249.22	12397.83	.80	.82	.95	>.001
4	12061.43	12249.15	.83	.82	.93	>.001
5	12021.13	12247.95	.81	.84	.94	>.001

Note: *N*=369; this table contrasts LPA models with different numbers of classes of pandemic scientists.

Table 2: Group assignments

	U.S. scientists		German scientists		All	
	n	*%*	*n*	*%*	*n*	*%*
Moderate Mainstreamers	97	48.5	69	40.8	166	45.0
Issue Advisors	50	25.0	56	33.1	106	28.7
Issue Advocates	37	18.5	16	9.5	53	14.4
Honest Brokers	16	8.0	28	16.6	44	11.9
All	200	100	169	100	369	100

Note: The table shows the distribution of U.S. and German pandemic scientists across the four groups identified through Latent Profile Analysis.

Table 3: Scale means for the identified groups

	Political involvement					
	U.S. scientists		German scientists		All	
	M	*SD*	*M*	*SD*	*M*	*SD*
Moderate Mainstreamers	4.71_{B}	.71	4.47_{B}	.86	4.61_{B}	.78
Issue Advisors	2.37_{A}	.75	2.36_{A}	.74	2.37_{A}	.73
Issue Advocates	5.86_{C}	.74	5.90_{C}	.73	5.87_{C}	.73
Honest Brokers	2.27_{A}	1.01	2.44_{A}	.89	2.38_{A}	.93
All	4.14	1.53	3.57	1.48	3.88	1.53
	Scientific neutrality					
	U.S. scientists		German scientists		All	
	M	*SD*	*M*	*SD*	*M*	*SD*
Moderate Mainstreamers	3.78_{B}	1.09	3.78_{B}	1.21	3.78_{B}	1.14
Issue Advisors	4.95_{C}	1.28	4.67_{C}	1.15	4.81_{C}	1.22
Issue Advocates	2.25_{A}	.97	1.63_{A}	.74	2.06_{A}	.95
Honest Brokers	5.35_{C}	1.04	$4.29_{B, C}$	1.46	4.67_{C}	1.41
All	3.92	1.48	3.96	1.46	3.94	1.47
	Epistemic authority					
	U.S. scientists		German scientists		All	
	M	*SD*	*M*	*SD*	*M*	*SD*
Moderate Mainstreamers	5.50_{B}	.89	5.41	$.75_{B}$	5.46_{B}	.83
Issue Advisors	5.71_{B}	.81	5.55	$.73_{B}$	5.62_{B}	.77
Issue Advocates	6.36_{C}	.54	6.25	$.61_{C}$	6.33_{C}	.55
Honest Brokers	3.38_{A}	.79	3.12	$.77_{A}$	3.21_{A}	.77
All	5.54	1.07	5.15	1.18	5.37	1.14

Notes: The table shows the mean answers of the participants to the three composite scores formed from the items answered by the respondents to the question "*How do you personally contribute to societal debates in your field?*" to access their self-images (1: do not agree at all; 7: absolutely agree). Different capitalized index letters indicate differences ($p<.05$) according to a Bonferroni-corrected post hoc test between the groups of pandemic scientists.

Supplemental Material 6: Descriptive results survey experiment

Table 1: Descriptive analysis, importance to communicate policy advice across scenarios

	U.S. scientists							
	Endemic		Pandemic		Control		Total	
	M	*SD*	*M*	*SD*	*M*	*SD*	*M*	*SD*
Moderate Mainstreamers	5.29_a	.81	4.93_a	.88	5.17_a	.80	5.14_B	.83
Issue Advisors	4.45_a	1.37	4.27_a	1.06	4.92_a	1.24	4.53_A	1.23
Issue Advocates	5.38_a	.95	4.27_a	1.09	5.51_a	.64	5.64_C	.82
Honest Brokers	4.92_a	1.31	4.00_a	1.15	5.20_a	1.02	$4.66_{A,B}$	1.21
	German scientists							
	Endemic		Pandemic		Control		Total	
Self-images	*M*	*SD*	*M*	*M*	*M*	SD	*M*	SD
Moderate Mainstreamers	5.43_a	.79	5.33_a	1.04	5.36_a	.53	$5.37_{A,B}$	.85
Issue Advisors	5.02_a	1.03	4.89_a	.99	5.14_a	.91	$5.04_{A,B}$	.95
Issue Advocates	5.00_a	.72	5.71_a	.71	5.90_a	.26	5.63_B	.68
Honest Brokers	4.43_a	1.16	4.84_a	1.12	5.14_a	1.03	4.84_A	1.09

Notes: n_1=199, n_2=169; the table shows the mean answers of the participants to the items that formed the composite score to access their willingness to communicate policy advice (1: very unimportant; 7: very important), as presented in my survey experiment. Different lowercase index letters mark a difference ($p<.05$) according to a Bonferroni-corrected post hoc test between the experimental groups. Different capitalized index letters indicate differences between the groups of pandemic scientists with different self-images.

Table 2: Descriptive analysis, importance to communicate policy advice across scenarios

	U.S. scientists							
	Endemic		Pandemic		Control		Total	
Containment measures	*M*	*SD*	*M*	*SD*	*M*	*SD*	*M*	*SD*
Inappropriate	5.03_a	1.13	4.82_a	1.16	5.13_a	.82	4.99_A	1.06
Appropriate	5.12_a	.94	5.00_a	1.12	5.24_a	.98	5.12_A	1.00
	German scientists							
	Endemic		Pandemic		Control		Total	
Containment measures	*M*	*SD*	*M*	*M*	*M*	SD	*M*	SD
Inappropriate	5.05_a	1.03	5.14_a	1.01	5.09_a	.85	5.11_A	.96
Appropriate	5.19_a	.81	5.28_a	1.08	5.55_a	.64	5.35_A	.85

Notes: n_1=199, n_2=169; the table shows the mean answers of the participants to the items that formed the composite score to access their willingness to communicate policy advice (1: very unimportant; 7: very important), as presented in my survey experiment. Different lowercase index letters mark a difference ($p < .05$) according to a Bonferroni-corrected post hoc test between the experimental groups. Different capitalized index letters indicate differences between the groups of pandemic scientists with different stances on containment measures.
